# Pilot-scale Production and Viability Analysis of Freeze-Dried Probiotic Bacteria Using Different Protective Agents

**DOI:** 10.3390/nu2030330

**Published:** 2010-03-11

**Authors:** Michele Savini, Cinzia Cecchini, Maria Cristina Verdenelli, Stefania Silvi, Carla Orpianesi, Alberto Cresci

**Affiliations:** 1 Synbiotec S.r.l., Via d’Accorso 30/32, 62032 Camerino, Italy; Email: michele.savini@studenti.unicam.it; 2 Dipartimento di Scienze Morfologiche e Biochimiche Comparate, Università di Camerino, Via Gentile III da Varano, 62032 Camerino, Italy; Email: cristina.verdenelli@unicam.it (M.C.V.); stefania.silvi@unicam.it(S.S.); carla.orpianesi@unicam.it(C.O.); alberto.cresci@unicam.it(A.C.)

**Keywords:** probiotics, prebiotics, cryoprotectans, freeze-drying, *Lactobacillus rhamnosus*, *Lactobacillus paracasei*

## Abstract

The functional food industry requires an improvement of probiotic strain stability during storage, especially when they are stored at room temperature. In this study, the viability of freeze-dried *Lactobacillus rhamnosus* IMC 501^®^ and *Lactobacillus paracasei* IMC 502^®^ using different protective agents (*i.e.*, glycerine, mannitol, sorbitol, inulin, dextrin, Crystalean^®^) was determined and compared with semi skimmed milk (SSM) control. No significant differences were observed between the tested protectants and the control (SSM) during storage at refrigerated conditions. During storage at room temperature, only glycerine was found to stabilize viability better than other tested substances.

## 1. Introduction

The primary role of the diet is to provide enough nutrients to meet metabolic requirements. Recent knowledge supports the hypothesis that, beyond meeting nutritional needs, diet may modulate various functions in the body and may play detrimental or beneficial roles in some diseases. Probiotic bacteria have a key role in achieving functional activity of foods [1]. 

In Europe, the rapid and continuing development of the human food and beverage probiotic market is one of the most prominent success stories in the functional foods industries [2]. The human gut naturally includes large numbers of intestinal bacteria species, but their composition is constantly changing. The numbers of natural bacterial species are constantly fluctuating with diet consumption, stress, oral antibiotic use, and they are also subjected to large fluctuations after intestinal infections. The consumption of oral probiotics acts to modify the intestinal microflora balance in a beneficial “re-balancing” manner, and thus helps the digestive health of the consumer. Probiotics are also known for their established benefits in improving gut disorders, such as ulcerative colitis, Crohn’s disease and irritable bowel syndrome. They are also beneficial in other conditions, such as heart disease, autism and allergies [3].

During recent years, the popularity and demand for non-milk-based probiotic formulations has increased. Buyers ask to extend the functional food market through the production and marketing of products different from milk and milk derivatives, in particular fruit juices, chocolate, cereals, ice cream and sausages are desired [4]. Furthermore, the availability of non-milk-based probiotic formulations is especially important for consumers who suffer from lactose intolerance or from milk-protein allergy. Thus, the final purpose is to setup probiotic strain production in a completely allergen free way [5].

Freeze-drying is a common method used to incorporate probiotics in foods. However, the viability of freeze-dried probiotic bacteria is affected during processing and storage. Freeze-dried probiotic organisms are protected by adding cryoprotectants, and the identification of protective agents that enhance cellular survival during storage and application in food is the key challenge [6].

The use of prebiotic substances in combination with probiotic strains (synbiotics) is an important topic for industrial purposes. In functional foods, prebiotic substances have a double role: they act to improve probiotic survival during storage in foods, and when reconstituted in the bowel, they act to enhance the digestive process [7]. Moreover, adding a prebiotic to a probiotic formulation increases the potential for the partnering probiotic strains to survive within the gastrointestinal tract of the host and the ability of the prebiotic to increase the number of beneficial bacteria directly itself once ingested by the host [8]. Therefore, the study of new synbiotic formulations is particularly anticipated [9].

The objectives of this research work were to perform a pilot-scale production of *Lactobacillus rhamnosus* IMC 501^®^ and *Lactobacillus paracasei* IMC 502^®^ and evaluate their viability during freeze-drying in presence of protective agents and prebiotics, and storage at different temperatures.

*L. rhamnosus* IMC 501^®^ and *L. paracasei* IMC 502^®^ are two human probiotic bacterial strains identified and characterized by Synbiotec research group (Synbiotec srl, Camerino, Italy). These strains are patented and experimentally documented [10,11].

## 2. Results and Discussion

### 2.1. Fermentations and Cell Yields

The fermentation of both strains was stopped after 19 h from inoculation. The final cell concentration was 5.7 × 10^8^ CFU/ml for *L. rhamnosus* IMC 501^®^ and 3.1 × 10^9^ CFU/ml for *L. paracasei* IMC 502^®^. 

The yield of *L. rhamnosus* IMC 501^®^ was 7.8 g of freeze-dried powder with a mean concentration of 3.1 × 10^11^ CFU/g (control sample). The yield of *L. paracasei* IMC 502^®^ was 7.2 g of freeze-dried powder with a mean concentration of 1.7 × 10^10^ CFU/g (control sample). Despite that the final concentration of the *L. rhamnosus* fermentative culture was lower than the *L. paracasei* one, the mean concentration of the *L. rhamnosus* freeze-dried powder was higher than that of *L. paracasei*.

The obtained cell concentrations after 19 h of fermentation process was higher than those obtained using static flask fermentation methods at controlled temperature (37 °C) (data not shown). The optimal time for fermentation inoculum was found to be between 4-5 h of flask growth for both *L. rhamnosus* IMC 501^®^ and *L. paracasei* IMC 502^®^.

### 2.2. Viability of Freeze-Dried Strains during Storage

**Figure 1:**
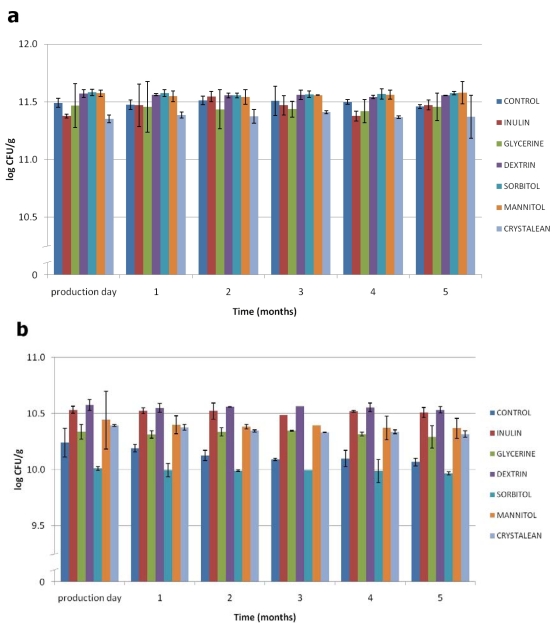
Viability of freeze-dried *L. rhamnosus* IMC 501^®^ **(a)** and *L. paracasei* IMC 502^®^ **(b)** bacteria using protective agents during storage at 4 °C for five months. Data are expressed as mean of triplicate ± standard deviation.

**Figure 2:**
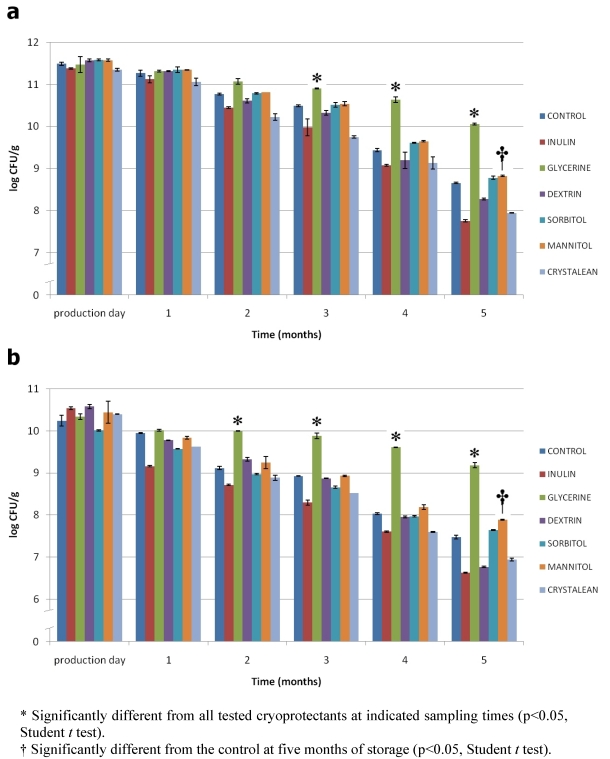
Viability of freeze-dried *L. rhamnosus* IMC 501^®^ **(a)** and *L. paracasei* IMC 502^®^ **(b)** bacteria using protective agents during storage at r.t. for five months. Data are expressed as mean of triplicate ± standard deviation.

The experimental results pertaining to survival of freeze-dried *L. rhamnosus* and *L. paracasei* during storage at room temperature (r.t.) and at 4 °C, using semi skimmed milk (SSM) as control and glycerine (Gly), inulin (In), dextrin (Dex), Crystalean^®^ starch (Crys), sorbitol (Sor) and mannitol (Man) as testing protective agents, are shown in Figure 1 and Figure 2. Survival of probiotic bacteria during the freeze-drying process was similar for all the tested cryoprotectant agents with respect to the controls. However, some differences were observed during storage at different temperatures.

During storage at 4°C (Figure 1), the viability of both strains was unchanged for the five months following production day, maintaining a constant trend, and there was no significant difference observed either among the tested protective agents or in comparison to the control. This indicates that the tested prebiotics and non-milk protectants have a cryoprotective function when added to harvested cell suspension before freeze-drying.

When the five months storage was at r.t. (Figure 2), the viability of both probiotic organisms decreased over the time, and some differences were observed. Among the tested substances, glycerine performed best in protecting the cells from storage injuries. This result suggests that the penetrating effect of glycerine may be more efficient than the non-penetrating oligosaccharides (inulin, dextrin, Crystalean^®^ starch) and control (SSM); furthermore, it is more active than the other tested polyalcohols (sorbitol and mannitol). In any case, mannitol provided a significant improvement of viability after five months of storage at r.t. with respect to the control (SSM) for both probiotic strains.

In this study, prebiotics were also included as protective agents. The cryoprotective effect of prebiotic substances needs to be deeply investigated. Capela *et al*. [12] evaluate the effect of prebiotics Raftilose^®^ (FOS), Hi-maize^®^ and Raftiline^®^ (inulin) on the viability of probiotic organisms in fresh and freeze-dried yogurt. They found that the presence of these oligosaccharides reduced the rate of cell death of the saccharolytic bacteria during storage, as prebiotics were available for their utilization. However, in freeze-dried yogurt, the viability of the probiotic organisms was not significantly lower than the control when prebiotics were added. On the basis of the obtained data, it is possible to hypothesize the use of prebiotic as cryprotectants when the storage of freeze-dried probiotics can be ensured at refrigerated conditions.

Although elucidation of cause-and-effect mechanisms still requires further investigation, it is important to remember that for each lactic acid bacteria strain of interest, the influence of various environmental conditions on survival in the dried state is specific for that species, and likely for that strain [13,14,15,16]. This work suggests that the cryoprotective action of the tested substances is similar for the two *Lactobacillus* species.

### 2.3. Evaluation of Molecular Stability during Storage

**Figure 3:**
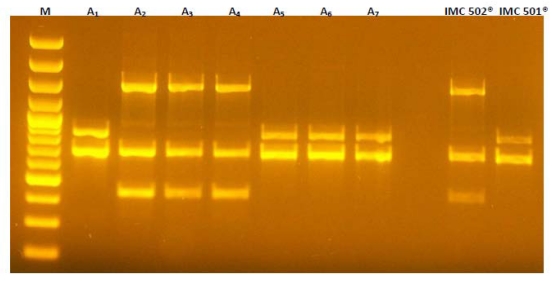
Detection of *L. rhamnosus* IMC 501^®^ and *L. paracasei* IMC 502^®^ by RAPD with primer M13. *Lane M* 100-bp DNA ladder; *lanes A1, A5, A6, A7* freeze-dried *L. rhamnosus* IMC 501^®^ samples; *lanes A2, A3, A4* freeze-dried *L. paracasei* IMC 502^®^ samples; *lane IMC 502^®^* reference strain *L. paracasei* IMC 502^®^; and *lane IMC 501^®^* reference strain *L. rhamnosus* IMC 501^®^.

Through RAPD-PCR it was possible to distinguish *L. rhamnosus* IMC 501^®^ from *L. paracasei* IMC 502^®^ and to confirm their strain identity profile. The RAPD profiles of the two *Lactobacillus* strains are shown in Figure 3. No contaminations were identified. Moreover, the application of this technique on reconstituted and cultivated freeze-dried probiotic strains confirmed the genetic stability of the two strains for the five months storage period.

The presence of *L. rhamnosus* IMC 501^®^ or *L. paracasei* IMC 502^®^ in the analyzed sample can be easily deduced by comparing the obtained profiles to standard ones, located in the last two lanes. As shown in Figure 3, DNA in lanes A2, A3, A4 are from *L. paracasei* IMC 502^®^ since they are identical to the corresponding reference strain *L. paracasei* IMC 502^®^ standard profile located in the next to last lane (*lane IMC 502^®^*), whereas DNA in lanes A1, A5, A6 and A7 are *L. rhamnosus* IMC 501^®^, identical to the corresponding standard profile located in the last lane(*lane IMC 501^®^*).

## 3. Experimental

### 3.1. Bacterial Strains

*L. rhamnosus* IMC 501^®^ and *L. paracasei* IMC 502^®^ were obtained from Synbiotec S.r.l., Camerino, Italy. The strains were stored in sterile glycerol at -80 °C and revived on MRS (deMann, Rogosa, Sharpe) broth medium (Oxoid S.p.A., Milan, Italy) overnight at 37 °C under aerobic conditions.

### 3.2. Pilot Scale Fermentation

For both *Lactobacillus* strains, the same protocol was adopted. Revived culture was inoculated in a 180 mL sterile MRS broth flask (10% inoculum) and incubated for 4 h at 37 °C. The 200 mL culture was then inoculated into 1.8 L MRS broth in a sterile glass autoclavable bioreactor (ADI Autoclavable Bio Reactor System 3 L, Applikon Biotechnology B.V., Schiedam, Netherlands). The fermenter was stirred at 110 rpm, the temperature was set at 37 °C and the pH controlled at 5.6 using NaOH 2.5 mol/L solution. Aeration was set at 0.5 L/min of sterile air and it was fed from the bottom part of the fermenter through a sparger pipe. The antifoam used was a siliconic type SILIFOOD 1600 (Silitex S.r.l., Cologna Veneta, VE, ITALY) at 0.1% (w/v). Sampling was conducted at 3 h, 6 h and 19 h after inoculation: each sample was analyzed for optical density at λ = 600 nm, Gram staining and bacterial plate counts. The fermentation process was stopped at 19 h when the stationary phase was reached.

### 3.3. Freeze-Drying

Cells were harvested by centrifugation at 4,000 g for 25 min at 4 °C (Thermo Scientific Heraeus Megafuge 1R Benchtop Centrifuge, Waltham, MA, USA) and poured in seven 100 mL flasks (5.6 ± 0.2 g in each flask). The cell mass was mixed with seven different protective agents in a ratio 1:5 (1 mL of cryoprotectant for 5 g of concentrated cells). Sterile suspension of protectants were prepared in phosphate buffered saline (PBS) solution at the concentration of 10% (w/v). The additives tested as protective agents (Table 1) were: inulin and dextrin (oligosaccharides); Crystalean^®^ starch (resistant starch); glycerine, sorbitol and mannitol (polyalcohols). Semi skimmed (SSM) commercially available was used as a control cryoprotectant. These protectants are all from Sigma-Aldrich (Sigma-Aldrich Srl, Milan, Italy) and approved as food additives by European Food Safety Authority (EFSA, Parma, Italy).

The 100 mL flasks were prepared as described and frozen at −80 °C in a static state for 24 h. Successively, samples were dried in a Zirbus freeze dryer (Zirbus Vaco 2, Bad Grund, DE) with a condenser temperature of −50 °C and a chamber pressure P < 0.08 mbar for 48 h.

### 3.4. Viability during Storage

Freeze-dried cultures were stored in portion-size sample sachets and under vacuum. They were placed at room temperature and at 4 °C to evaluate the stability. 

The viability of the freeze-dried samples was performed through plate count technique at production day and over the five months of storage. The bacterial counts of each sample were compared with the control sample at each time.

**Table 1:** Substances used as cryoprotectants.

Substances	Chemical nature	Prebiotic action
Inulin (In)	Oligosaccharide	yes
Dextrin (Dex)	Oligosaccharide	yes*
Crystalean® starch (Crys)	Resistant starch	yes*
Glycerine (Gly)	Polyalcohol	no
Sorbitol (Sor)	Polyalcohol	no
Mannitol (Man)	Polyalcohol	no

* the prebiotic properties are still under investigation.

### 3.5. Statistical Analysis

Statistical significance of the differences in survival of the freeze-dried cells from the different production condition (testing protectants *versus* control, room temperature *versus* refrigerated storage) during freeze drying and five months storage was analyzed by t-test (paired, one tailed). A p-value < 0.05 was considered statistically significant.

### 3.6. Molecular Stability during Storage

Molecular stability of the probiotic strains was determined by Randomly Amplified Polymorphic DNA (RAPD). RAPD was performed with the following random primers:

M13 minisatellite core sequence (5’-GAGGGTGGCGGTTCT-3’), RP (5’-CAGCACCCAC-3’) and R5 (5’-AACGCGCAAC-3’). Reactions were carried out in 25 μL amplification mixtures with 12.5 μL of 2x Master Mix (Fermentas, Burlington, Canada), 0.5 μL of 1 μM primer, 1 μL of DNA (approximately 50 ng) and 11 μL of water. The reaction mixtures with M13 primer, after incubation at 94 °C for 2 min, were cycled through the following temperature profile: 30 cycles 94 °C for 60 s, 42 °C for 20 s and 72 °C for 2 min, with a final extension at 72 °C for 10 min. The primer RP was used under the following amplification conditions: one cycle 94 °C for 3 min, 45 °C for 45 s, 72 °C for 1 min; 30 cycles 94 °C for 45 s, 45 °C for 45 s, 72 °C for 1 min; one cycle 94 °C for 45 s, 45 °C for 45 s, 72 °C for 5 min. The reaction mixtures with R5 primer, after incubation at 94 °C for 5 min, were cycled through the following temperature profile: 40 cycles 94 °C for 60 s, 29 °C for 90 s and 72 °C for 2 min, with final extension was carried out at 74 °C for 5 min. The PCR was conducted in a Tpersonal Thermal Cycler (Biometra). Amplification products were separated on a 2% agarose gel containing 0.5 μg/mL (w/v) of ethidium bromide (GIBCO BRL).

## 4. Conclusions

Two probiotic *Lactobacillus* strains were fermented in a pilot bioreactor controlling pH (5.6), temperature (37 °C), stirrer (110 rpm) and aeration (0.5 L/min), over the duration of the process to optimize the biomass yield. The obtained cell concentrations after 19 h of fermentation process yield was higher than those obtained using static flask fermentation methods at controlled temperature. 

Alternative protective agents are applicable to freeze-drying process to obtain high survival of cells. During storage at room temperature, only glycerine was found to stabilize viability better than other tested substances. Mannitol provides a significant improvement of viability after five months of storage at room temperature with respect to the control for both probiotic strains. The cryoprotective action of tested substances is similar for the two *Lactobacillus* species. It is possible to suppose the use of prebiotics as cryprotectants when the storage of freeze-dried probiotics at refrigerated conditions can be ensured.
